# Assessment of pediatric breast ultrasound less is more: a practical imaging approach

**DOI:** 10.1177/02841851241287924

**Published:** 2024-10-15

**Authors:** Elisabetta Giannotti, Rachel Sun, Nuala Healy, Fleur Kilburn-Toppin, Carmelo Sofia, Andrew HS Lee, Maria Adele Marino

**Affiliations:** 1 Cambridge Breast Unit, 2153 Cambridge University Hospital NHS Foundation Trust, Cambridge, UK; 2 Nottingham Breast Institute, 9820 Nottingham University Hospitals NHS Trust, Nottingham City Hospital, Nottingham, UK; 3 Department of Radiology, 57978 Beaumont Hospital, Dublin, Ireland; 4 Department of Radiology, Royal College of Surgeons in Ireland, Dublin, Ireland; 5 Department of Biomedical Sciences and Morphologic and Functional Imaging, Policlinico Universitario “G.Martino”, 18980 University of Messina, Messina, Italy; 6 Histopathology Department, 9820 Nottingham University Hospitals NHS Trust, City Hospital Campus, Nottingham, UK

**Keywords:** Breast, breast cancer, ultrasound, pediatric

## Abstract

**Background:**

Breast cancer in pediatric patients is rare, but ultrasound (US) is widely utilized for symptomatic cases.

**Purpose:**

To determine biopsy and cancer detection rates of pediatric patients and to assess if breast US can be omitted.

**Material and Methods:**

A retrospective review of a 5-year period was conducted of single-center breast US performed in patients aged <19 years. Data regarding presentation, clinical opinion (P1–5 score), and US (U1–5 score) were collected. If biopsy or surgery was performed, pathology was reviewed (B1–5 score).

**Results:**

In total, 579 patients were included (19 boys, 560 girls; mean age=16.2±1.9 years; age range=0–18 years). Clinical examination was normal or benign (P1/P2) in all boys (100%) and 557/560 (99.5%) girls, and P3 in 3 (0.5%) girls. Of US, 52% demonstrated normal findings (U1) for both sexes (300/579); in the remaining cases, the most frequent findings were gynecomastia in 12/19 boys and well-defined breast masses in 208/560 girls. Of the 560 girls, 6 (1%) underwent US-guided biopsy, with final histology of fibroadenoma (B2) in all cases, while 27 (5%) had a surgical excision, with final histology of fibroadenoma (22/27, 81.5%), hamartoma (2/27, 7.4%), benign phyllodes tumor (2/27, 7.4%), and angiomyxoma skin lesion (1/27, 3.7%). No malignant lesions were diagnosed at the time of clinical referral or during the 18-month follow-up in patients with a well-defined mass on US.

**Conclusion:**

Breast malignancy is extremely rare in pediatric population. US can be safely omitted if clinical examination is normal; this approach would have avoided breast US in 52% of patients in this study.

## Introduction

While breast cancer is rare in the pediatric population, breast symptoms are common in this age group. The overwhelming majority of breast symptoms in children and adolescents are self-limiting (1,2) but often cause anxiety and significant family distress. Approximately 10% of symptomatic referrals to the breast clinic are adolescents aged <18 years, of which <1% are aged <10 years (3). The spectrum of breast disease in the pediatric population is different from that in adults, and almost all lesions are benign, with the reported prevalence of breast cancer in girls/women under the age of 20 years well below 0.1 per 100,000 (4).

As there is a low prevalence of breast cancer in the pediatric and adolescent population, a tailored approach to its diagnosis and management is mandatory for breast complaints in this group, given the risk of injury to the developing breast secondary to biopsy and intervention (1). Clinical evaluation is an essential component in the assessment of pediatric breast complaints. With a pertinent history and physical examination, many pediatric breast complaints can be correctly categorized and managed with reassurance only, limiting imaging workup to a small minority. When necessary, ultrasound (US) is the primary imaging modality of choice, given its diagnostic specificity and lack of ionizing radiation (5). In contrast to the adult population, mammography is contraindicated in the pediatric population due to the extremely low risk of breast cancer (6), the increased risk of radiation-induced malignancy (7), and the poor image quality due to dense fibro-glandular breast tissue.

Varying referral pathways are in place to diagnose and treat children and adolescents with breast symptoms. However, a number of these, including the Association of Breast Surgery (ABS) guidelines, suggest referral to a one-stop breast clinic (cancer pathway) for breast symptoms, which can cause unnecessary parental and child anxiety, especially given the overwhelming likelihood of benign etiology (3). Referral to a symptomatic breast unit, which comprises a significantly older population cohort, is far from ideal, and in most other instances, pediatric patients are reviewed in a pediatric setting. The aim of this study was to review the outcomes of pediatric patients who underwent breast ultrasound in Nottingham Breast Institute, Nottingham University Hospital, Nottingham, UK over a 5-year period, to determine biopsy rates, cancer detection rates, and to elucidate if breast ultrasound can be safely omitted.

## Material and Methods

The study was registered as a service evaluation and approval was obtained from the Quality and Safety Information System Clinical Audit Team. As this was an observational study without any intervention, formal ethics approval and individual patient consent was not deemed necessary.

A retrospective review was undertaken of all patients aged <19 years at the time of attendance who had a breast ultrasound performed in a single center between January 2016 and December 2020.

### Patients

Patients, both male and female, aged <19 years, who had received a breast clinical examination and a breast ultrasound at Nottingham Breast Institute, Nottingham University Hospital between January 2016 and December 2020 due to a breast-related concern were included in the study. The exclusion criteria included cases with incomplete data on examination, imaging, or outcomes, as well as those with less than 18 months of follow-up.

Data regarding clinical presentation, clinical examination score, and ultrasound findings were collected. If biopsy or surgery was performed, pathology was reviewed. At least 18 months of follow-up was achieved for all patients. Patients were referred from primary care or pediatrics to a symptomatic breast clinic. All patients underwent clinical breast examination by a consultant surgeon, surgical trainee, or nurse practitioner in the breast unit. Patients were given a P score for their clinical examination, based on clinical suspicion, using the UK ABS 5-point classification, where P1 is normal, P2 is benign, P3 is indeterminate, P4 is suspicious for malignancy, and P5 is considered highly suspicious for malignancy (8). Patient electronic hospital records and the patient archiving and communication system (PACS) were reviewed to establish patient demographics, clinical examination findings, imaging performed, biopsies performed, pathology findings, and eventual patient outcomes. Data were retrospectively collected by a breast radiologist and a breast advanced practitioner.

### Imaging workup

All patients included in the study had clinical examination and subsequent ultrasound performed.

Mammography was not performed in this age group. Ultrasound was performed by a breast radiologist or advanced breast practitioner, all sub-specialized in breast imaging with previous dedicated training and a range of 3–23 years of experience in breast imaging. All breast ultrasound examinations were performed with knowledge of clinical findings. Ultrasound was allocated a U score, using the Royal College of Radiology 5-point imaging classification using the Stavros criteria, where U1 is normal, U2 is benign, U3 is indeterminate, U4 is suspicious for malignancy, and U5 is highly suspicious for malignancy (9,10). Breast ultrasound was performed using a high-frequency linear array transducer (15–18 MHz) (Siemens Medical Solutions, Erlangen, Germany).

Ultrasound-guided core biopsy (CB) was carried out when considered necessary by the radiologist performing the ultrasound, according to sonographic suspicion. Solid breast masses were assessed for benign and malignant features utilizing the Stavros criteria (9,10).

Surgical excision was performed when considered necessary by the consultant surgeon, based on clinical findings. All cases where biopsies or surgical excision was undertaken were discussed at the breast multidisciplinary meeting. CB/results were categorized as B1–B5 as recommended in UK guidelines (11).

### Statistical analysis

Statistical analyses were performed using SPSS version 22.0 (IBM Corp., Armonk, NY, USA). Descriptive statistics were calculated for patients’ demographic and clinical characteristics. Frequencies and percentages were used to summarize the distribution of histology.

## Results

A total of 579 children and adolescents (19 boys, mean age = 16.6 ± 1.3 years, age range = 14–18 years; 560 girls, mean age = 15.7 ± 1.9 years, age range = 0–18 years) underwent breast ultrasound. Two of the study group were children (age <12 years; mean age = 0.5 years; age range = 0–1 years) and the remaining 577 were adolescents (age 12–18 years; mean age = 16.4 ± 1.9 years; age range = 10–18 years). The 579 patients were reviewed in a specialist breast clinic; of them, 574 were referred from primary care and five were referred by a pediatrician.

Table 1 demonstrates the presenting symptoms of the pediatric and adolescent patients who underwent ultrasound. In both sexes, the most common complaint was a breast lump, accounting for 94% in girls and 95% in boys.

**Table 1.** Distribution and frequency of reason for clinical referral, stratified for both sexes.

Reason for referal		
Symptom	Male (n = 19)	Female (n = 560)
Lump	18 (95)	524 (93.6)
Infection	1 (5)	23 (4.1)
Nipple discharge	-	6 (1.1)
Lump and skin changes	-	4 (0.7)
Trauma	-	3 (0.5)

Values are given as n (%).

### Clinical examination

Clinical examination scores for girls were as follows: P1 = 290 (52%); P2 = 267 (47.5%); and P3 3 (0.5%). The examination scores for boys were as follows: P1 = 8 (42%) and P2 = 11 (58%). A P4 or P5 score was not allocated for either sex, and no P3 scores were encountered in boys.

All P3 lesions were encountered in female adolescents and they all corresponded to well-defined breast masses on ultrasound in keeping with benign fibroadenomas and were reassured and discharged.

### Ultrasound

The imaging features on ultrasound of all patients are presented in Table 2. Two children (aged <12 years) presented with a breast lump, and the imaging findings were diagnostic of benign entities – a lipoma and a breast bud, respectively. Ultrasound was normal in both sexes in 300/577 (52%) adolescents.

**Table 2.** Ultrasound imaging findings, stratified for both sexes, with distribution and frequency.

Ultrasound imaging findings	Male (n = 19)	Female (n = 560)
Normal	6 (31.6)	294 (52.5)
Gynecomastia	12 (63.1)	-
Fluid collection (abscess/hematoma)	1 (5.3)	11 (2)
Well-defined mass (likely fibroadenoma)	-	208 (37.1)
Cyst	-	25 (4.5)
Skin lesion	-	8 (1.4)
Ductal ectasia	-	3 (0.5)
Lymph node	-	3 (0.5)
Breast buds	-	3 (0.5)
Lipoma	-	2 (0.4)
Hamartoma	-	2 (0.4)
Pregnancy adenoma	-	1 (0.2)

Values are given as n (%).

Of the patients, 52% had a normal clinical examination (P1; 298/579) and ultrasound did not show any relevant pathology. The clinical score of all patients and corresponding imaging score and ultrasound findings are presented in Table 3. More detailed information of clinical symptoms, ultrasound score, and findings in relation to the P clinical score is described in Tables 4–8.

**Table 3.** Clinical score (P) in male and female patients and their ultrasound (U) score, and findings.

P score	Boys/girls	U score
P1 Normal (n = 298)	8/290	U1 Normal (292) U2 Fluid collection (2) U2 Gynecomastia (2) U2 Ductal ectasia (1) U2 Hamartoma (1)
P2 Benign (n = 278)	11/267	U2 Well-defined mass (205) U2 Cyst (25) U2 Gynecomastia (10) U2 Fluid collection (10) U1 Normal (8) US Sebaceous cyst (8) U2 Breast buds (3) U2 Lymph node (3) U2 Ductal ectasia (2) U2 Lipoma (2) U2 Pregnancy adenoma (1) U2 Hamartoma (1)
P3 Indeterminate (n = 3)	0/3	U2 Well-defined mass (3)

Values are given as n.

**Table 4.** Clinical P1 findings in boys (n = 8) and their clinical symptoms, ultrasound (U) score, and findings.

Reason for referral/symptom	US score and findings
Lump (8)	U1 Normal (6) U2 Gynecomastia (2)

Values are given as n.

**Table 5.** Clinical P2 findings in boys (n = 11) and their clinical symptoms, ultrasound (U) score, and findings.

Reason for referral/symptom	US score and findings
Lump (10)	U2 Gynecomastia (10)
Infection (1)	U2 ABSCESS (1)

Values are given as n.

**Table 6.** Clinical P1 findings in girls (n = 290) and their clinical symptoms, ultrasound (U) score, and findings.

Reason for referral/symptom	US score and findings
Lump (271)	U1 Normal (269) U2 Ductal ectasia (1) U2 Hamartoma (1)
Infection (12)	U1 Normal (11) U2 Abscess (1)
Nipple discharge (2)	U1 Normal (2)
Lump and skin changes (2)	U1 Normal (2)
Trauma (3)	U1 Normal (2) U2 Hematoma (1)

Values are given as n.

**Table 7.** Clinical P2 findings in girls (n = 267) and their clinical symptoms, ultrasound (U) score, and findings.

Reason for referral/symptom	US score and findings
Lump (250)	U1 Normal (4) U2 Breast buds (2) U2 Lymph node (3) U2 Abscess (4) U2 Well-defined mass (202) U2 Cyst (22) U2 Sebaceous cyst (8) U2 Lipoma (2) U2 Pregnancy adenoma (1) U2 Ductal ectasia (1) U2 Hamartoma (1)
Infection (11)	U1 Normal (4) U2 Abscess (5) U2 Cyst (2)
Nipple discharge (4)	U2 Well-defined mass (2) U2 Ductal ectasia (1) U2 Cyst (1)
Lump and skin changes (2)	U2 Breast buds (1) U2 Well-defined mass (1)

Values are given as n.

**Table 8.** Clinical P3 findings in girls (n = 3) and their clinical symptoms, ultrasound (U) score, and findings.

Reason for referral/symptom	US score and findings
Lump (3)	U2 Well-defined mass (3)

Values are given as n.

In 175 cases, the sonographic features were considered benign and the patient was reassured and discharged. Patient data records were reviewed for these 175 patients, with no evidence of malignancy within at least 18 months after the initial visit.

### Biopsy, surgical excision, and follow-up

**Fig. 1.**
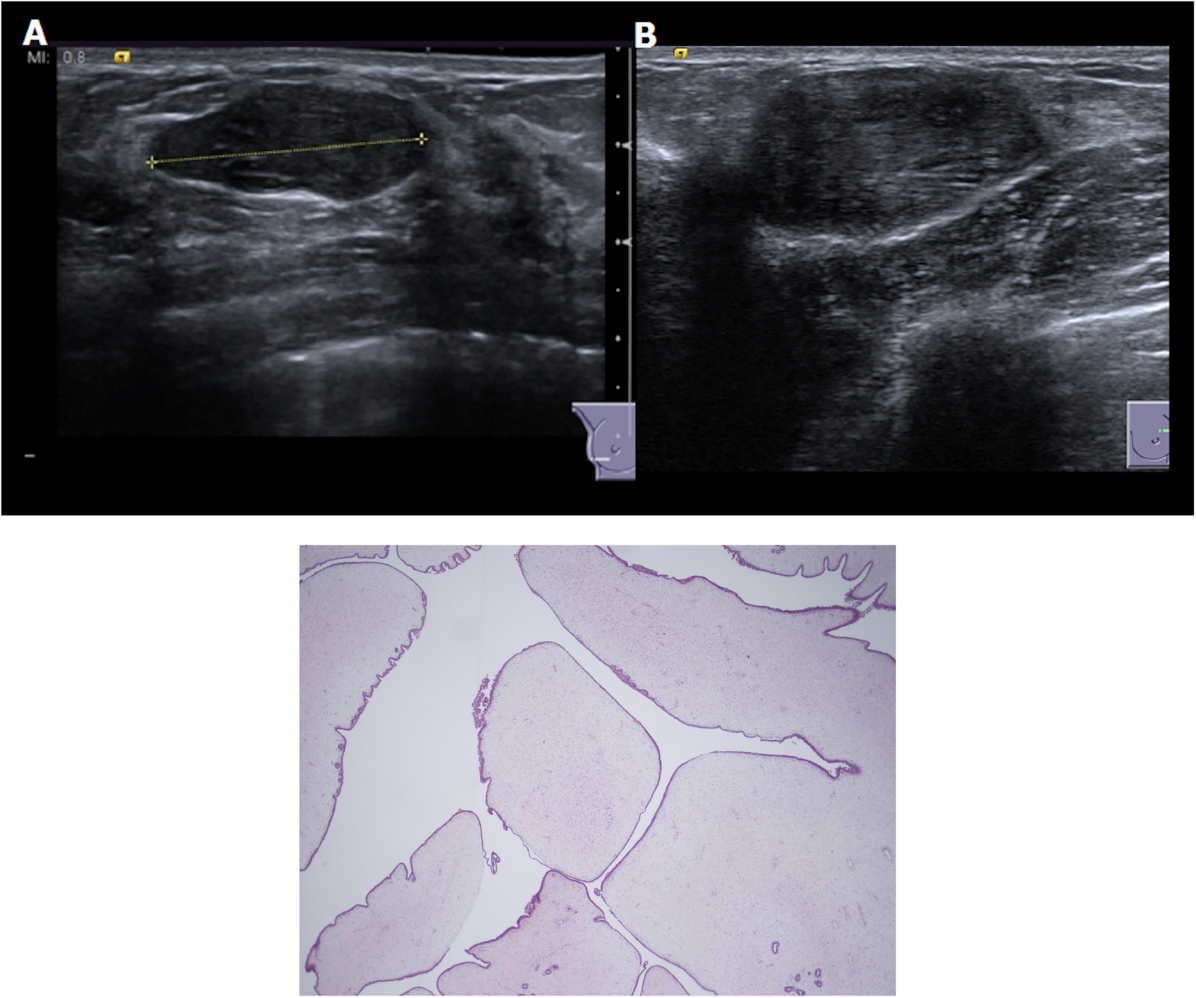
(A, B) B-mode ultrasound demonstrates in two circumscribed hypoechoic oval masses with parallel orientation and no posterior features associated. Both lesions were classified as U2 and the final histology revealed the diagnosis of benign phyllodes tumor. (c) Pathological images of a phyllodes tumor showing low power leaf-like architecture and expansion of the stroma.

Of the 579 patients, 6 (1%) underwent ultrasound-guided biopsy with final histology confirmed as fibroadenoma in all cases; 27/579 (4.8%) patients underwent a surgical excision with a final histology of fibroadenoma (22/27, 81.5%), hamartoma (2/27, 7.4%), benign phyllodes tumor (2/27, 7.4%) and angiomyxoma of the skin (1/27, 3.7%). Both phyllodes tumors (2/579; 0.3%) were histologically benign and were clinically palpable and classified as benign P2 (Fig. 1). The phyllodes tumors did not undergo CB before surgical excision and were excised due to their size (>3 cm).

No malignant lesions were identified in the study group of 579 children and adolescents, at the time of initial clinical referral and ultrasound, or within 18 months of referral based on review of patient records.

## Discussion

This study is an evaluation of the outcomes of a large group of pediatric patients (n = 579) attending with a breast complaint. The study highlights that when breast clinical examination is normal (298/579, 52%), no concerning pathology was present on breast ultrasound. Of those patients who underwent biopsy (6/579) or surgical excision (27/579), final histology was deemed benign. No cancers were detected on biopsy, surgical excision, or within the 18 months of follow-up. Therefore, even in the presence of a benign palpable lump, the risk of malignancy is extremely low with no requirement for CB before surgical excision. Based on our findings, breast biopsy and surgery could be avoided in the pediatric population, and reserved for only select pediatric cases after discussion with the multidisciplinary team.

Breast imagers play a crucial role in identifying benign findings on breast ultrasound as they perform the final step in the workup process, deciding if further follow-up or biopsy is necessary. It is essential that breast/pediatric imagers and clinicians are educated about normal breast development and the spectrum of pediatric breast pathology. In addition, they should be made aware that imaging in cases of normal clinical examination is of extremely low yield. As determined in this study, pediatric breast complaints can be correctly categorized as a normal developmental process or physiological changes, and in the vast majority of cases no imaging or intervention is required.

There is relatively limited literature on pediatric breast imaging (12–18), largely due to the extremely low rates of malignancy in patients aged <19 years, with an estimated annual age adjusted incidence well below <0.1 cases per 100,000. Despite this, increasing media attention on breast cancer and increasing breast awareness among young women, combined with possible unwillingness or lack of success in reassuring these adolescents or their parents in primary care, may be reasons for young women being referred for specialist opinion.

In our study, the most frequent reason for referral was a lump for both boys (95%) and girls (94%). This is in agreement with the study by Durmaz et al. (12), which identified that the most frequent reasons for referral to radiology were a palpable mass and gynecomastia. Hart et al. (13) demonstrated that the most frequent reasons for breast referral in children and adolescents were premature thelarche and asymmetric breast enlargement in children.

**Fig. 2.**
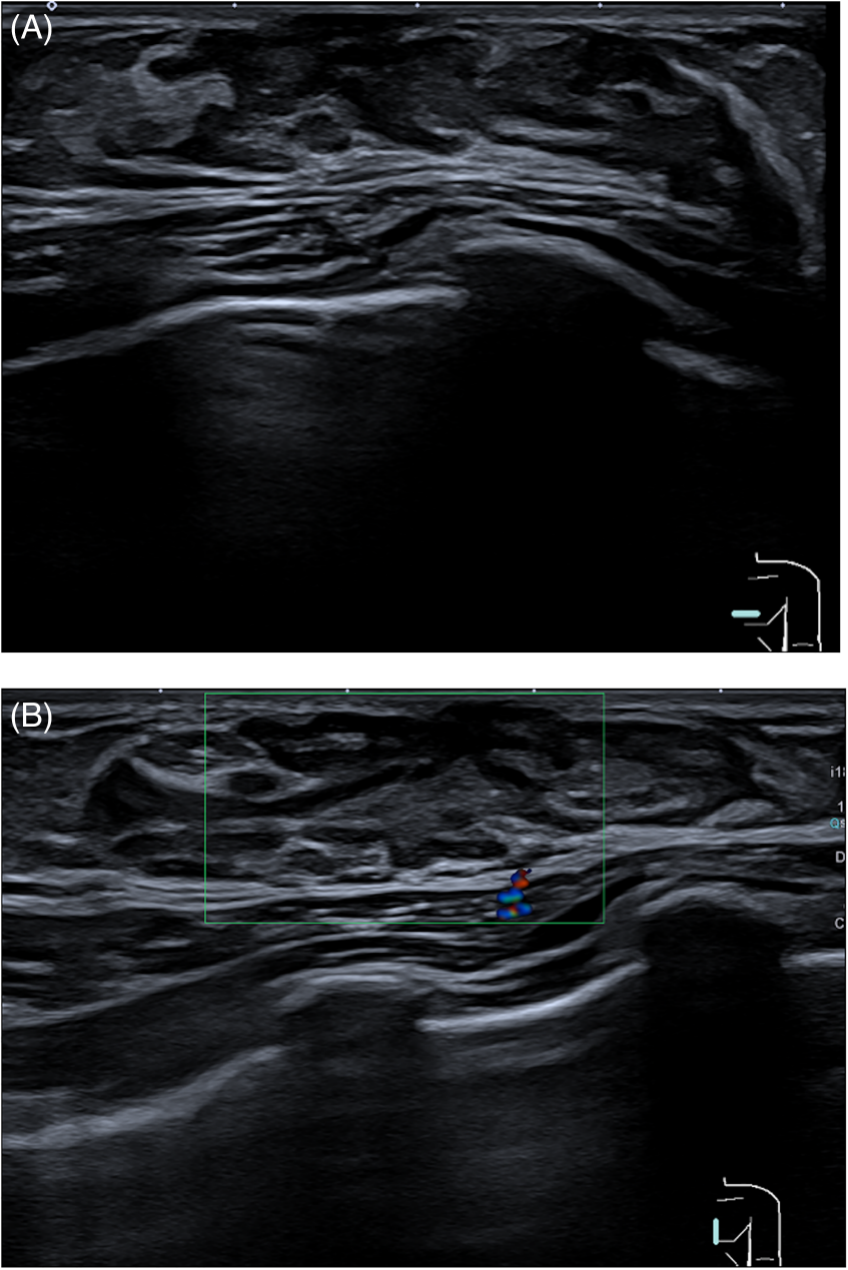
(A) B-mode and (B) color Doppler ultrasound demonstrates retroareolar hypoechoic tissue relative to fat with absence of blood flow in keeping with breast buds.

For the radiologist, it is very important to be familiar with normal breast development and possible imaging findings, as normal anatomic structures can mimic breast masses. In neonates aged 6–12 months, bilateral palpable sub-areolar nodules are due to enlargement of prepubertal breast ducts, due to maternal hormonal influence. Premature thelarche or onset of unilateral breast development can also be the cause of a palpable lump. Ultrasound has a pivotal role in demonstrating breast buds and can be utilized to reassure the patient (Fig. 2).

**Fig. 3.**
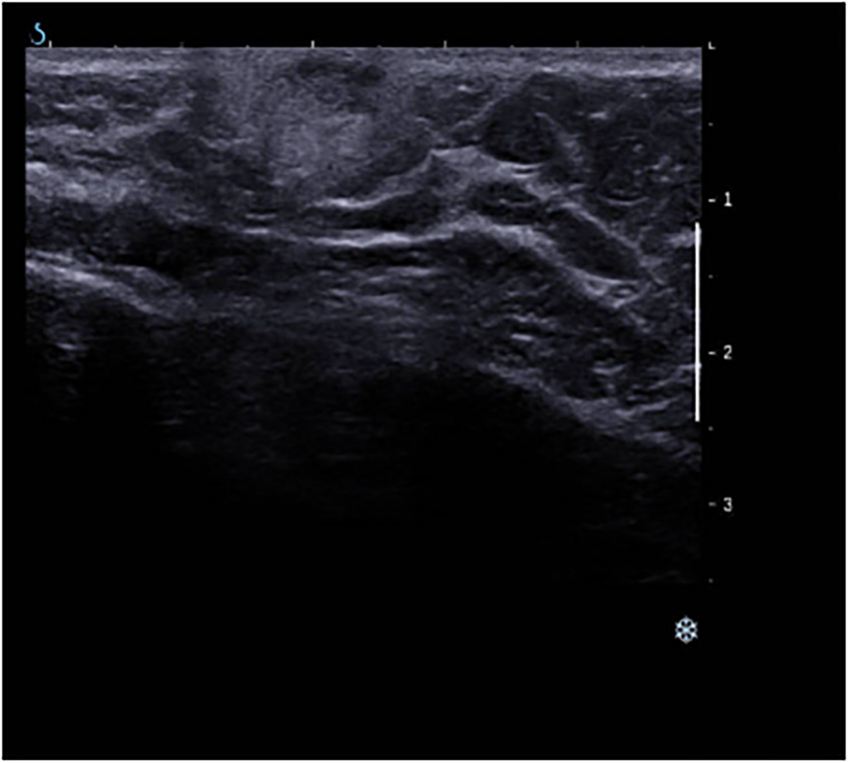
B-mode ultrasound demonstrates superficial circumscribed hypoechoic oval masses with parallel orientation within the skin with associated hyperechoic rim in keeping with an infected sebaceous cyst.

It is also important for radiologists to be aware of the possible benign entities encountered on pediatric ultrasound, such as inflammatory lesions (mastitis and abscess), benign neoplastic lesions (fibroadenoma, juvenile fibroadenoma if >50 mm, hamartoma, and hemangioma), benign non-neoplastic entities such as cysts, lymph nodes, hematomas, galactoceles, and skin lesions (Fig. 3).

Children and adolescents can also present with bloody nipple discharge due to drugs, exercise, or trauma; a conservative approach is recommended as it is usually a self-limiting event.

**Fig. 4.**
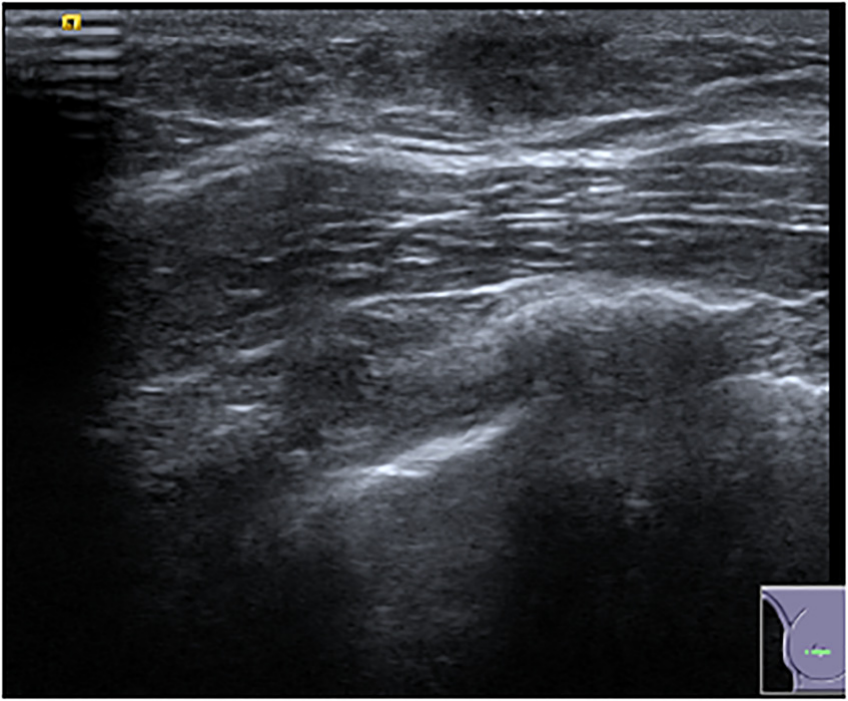
B-mode ultrasound shows retroareolar triangular, hypoechoic disc-shaped nodule in keeping with gynecomastia.

In our study, ultrasound was normal in 52.5% of girls and showed a well-defined mass in 37% or a cyst in 4.5%. Five of these cysts underwent aspiration; however, cysts are benign non-neoplastic findings that do not need to be aspirated. Durmaz et al. (12) showed natural regression in follow-up of cystic lesions that resolve over time. In boys, 31.6% of ultrasound examinations performed were normal, with gynecomastia being the most frequent finding in 52.5%. Gynecomastia is a frequently occurring, self-limiting finding encountered in this group, presenting in 90% of male neonates due to maternal hormones and in 75% of boys (13) (Fig. 4).

Our findings are in agreement with those of a previous study by Hart et al. (13) where the most frequent radiologic findings were gynecomastia in boys and normal glandular tissue in girls. That study also did not detect any malignancy in their pediatric population, and fibroadenoma was the most frequent solid mass in children aged >8 years. Fibroadenoma is the most frequent benign neoplastic lesion in adolescents. There is a significant overlap in imaging findings between fibroadenomas and phyllodes. Although some characteristics of phyllodes include intralesional cysts and clefts, they have a low specificity and surgical excision is advised for any rapidly growing mass in the adolescent breast even if it has been previously characterized as benign by CB (19). This is because the possibility of phyllodes underestimation diagnosed at CB is approximately 20% (19). To exclude the possibility of a phyllodes tumor, removal should be considered if a fibroadenoma is rapidly growing, there is persistent discomfort and pain, despite biopsy indicating fibroadenoma, and according to Hubbard et al., an immobile poorly defined mass larger than 25 mm (19). A recent study defined a significant growth (increase in volume >15%/month) as a risk factor of phyllodes tumor (20).

Our study confirms that breast cancer is extremely rare in the pediatric population, with no invasive malignancy identified in our population. All phyllodes tumors were clinically palpable and classified as benign on histology, did not receive a preoperative biopsy, and were managed clinically due to their size (>30 mm). According to our findings, ultrasound in children and adolescents could have been safely omitted if clinical examination was normal.

The present study has some limitations, including its retrospective nature and the study population from a single center. A future multicenter study with a larger patient population could validate the results of our study. We do not have information on pediatric patients referred to the breast unit who did not have imaging performed and were discharged after clinical examination. Clinical follow-up in our study was up to 18 months and a longer clinical follow-up might be helpful in assessing the long-term outcomes of breast lesions in the pediatric population. Finally, only a small subset of the patient population had a pathological diagnosis of the breast lesions in our study. A future study focusing on imaging findings and pathological diagnoses of breast lesions in the pediatric population could further illustrate the nature of breast lesions seen in this population.

As radiologists, it is important to discuss with clinicians the role and usefulness of ultrasound in pediatrics and adolescent patients. If clinical examination is normal, this group of patients could potentially be managed clinically only, without ultrasound, as illustrated in this study. A multidisciplinary approach should be employed, with discussion between clinician and radiologists in challenging cases, with ultrasound often performed in difficult cases. If there are clinical concerns regarding infection, inflammation or non-neoplastic pathology, an ultrasound should be performed. Radiologists should be familiar with benign pathology in the pediatric population and be confident in categorizing imaging findings as benign, thereby eliminating the necessity for additional follow-up or intervention, especially when ultrasound reveals reassuring features.

In conclusion, although children and adolescents are a small proportion of overall referrals to breast units, they can generate significant imaging workload. Referrals to a predominantly adult breast unit is far from ideal and are often a cause of significant patient and parental anxiety. Breast cancer is extremely rare in this population, and we propose that ultrasound could safely be omitted if clinical examination is normal. Adopting this approach would have avoided breast ultrasound and thereby breast clinic referral in 52% (298) of patients. Modification of how pediatric patients are managed in order to minimize stress and anxiety for patients and their families should be considered. One potential solution is that they could be managed in a dedicated clinic in the pediatric department, jointly run by a breast surgeon/clinician and a radiologist, eliminating the need for ultrasound in cases of normal clinical examination.
